# Successful resection of a giant well-differentiated liposarcoma using a modified snare technique

**DOI:** 10.1055/a-2895-3621

**Published:** 2026-06-30

**Authors:** Ying Peng, Chuanfang Chen, Zhenyu Wu, Sha Gu, Juan Fan, Xiaofeng Feng

**Affiliations:** 1 Department of Gastroenterology 388288 Southwest Hospital, Third Military Medical University (Army Medical University)ChongqingChina

**Fig. 1:**
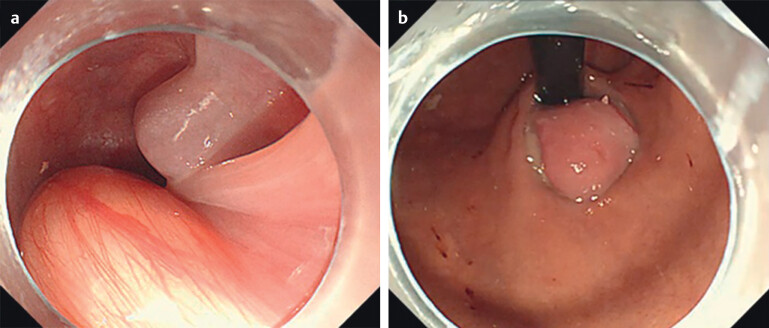
Preoperative endoscopic views. ( **a** and **b** ) A 21-cm giant pedunculated lesion occupies the esophageal lumen, extending from the esophageal inlet down to the gastric cardia.

**Fig. 2:**
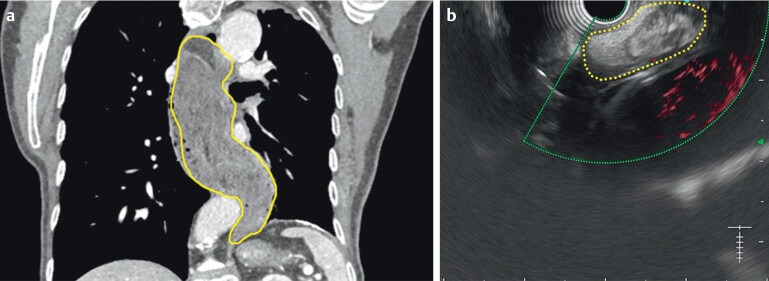
Preoperative computed tomography (CT) and endoscopic ultrasonography (EUS) evaluation. ( **a** ) A giant soft-tissue mass (yellow outline) occupied the esophageal lumen and extends to the gastroesophageal junction. ( **b** ) The mass (yellow dashed outline) displayed heterogeneous echogenicity, featuring well-demarcated mixed-echoic structures within predominant hyperechoic areas and scattered calcifications.

A 75-year-old woman presented with progressive dysphagia and was subsequently admitted to our hospital. Gastroscopy revealed a giant 21-cm lesion originating from the esophageal inlet and prolapsing through the cardia ( Fig. 1a–b ). Preoperative computed tomography and endoscopic ultrasonography identified a massive intraluminal mass with well-demarcated mixed-echoic structures embedded within predominant hyperechoic areas and scattered calcifications, highly suggestive of a liposarcoma ( Fig. 2a–b ). Following a preoperative discussion, endoscopic resection was planned.

**Fig. 3:**
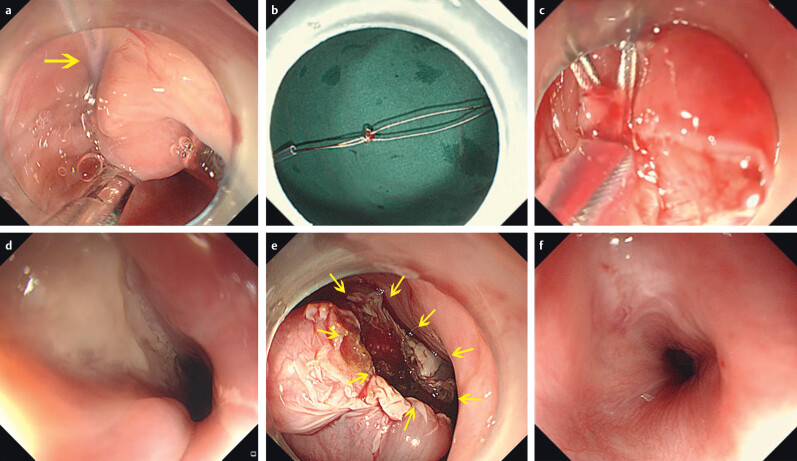
Procedural steps of the modified snare technique and follow-up. ( **a** ) Forceps-assisted traction guided the snare to optimize exposure, confirming a broad pedicle base at the postcricoid region (the yellow arrow indicated the snare sheath). ( **b** ) Two conventional snares were intertwined. ( **c** ) A third snare was applied to constrict the coupled snares. ( **d** ) A macroscopic view of the excised specimen (the yellow arrow indicated the broad pedicle base). ( **e** ) The mucosal defect at the postcricoid region. ( **f** ) Gastroscopy at 3 months confirmed complete mucosal recovery with a stable scar.

**Fig. 4:**
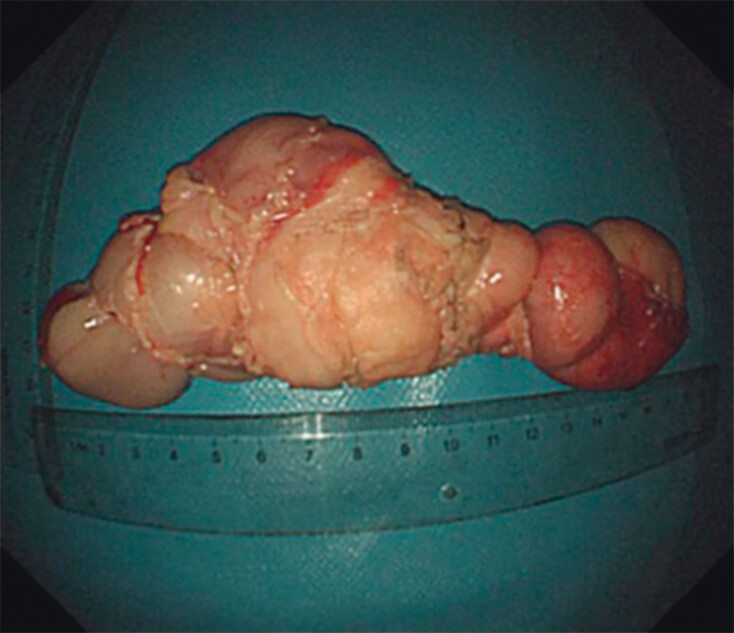
Specimen reconstruction. The piecemeal specimens were reconstructed ex vivo to verify the original 21-cm morphology.

During the procedure, forceps-assisted traction was employed to guide the snare and optimize exposure, definitively confirming a broad pedicle base at the postcricoid region ( Fig. 3a ). Although the lesion was pedunculated, the narrow lumen and broad pedicle posed a significant risk of anatomical disorientation during conventional endoscopic submucosal dissection (ESD). To encompass the broad base, we applied a modified snare technique. Two conventional snares were intertwined to form a larger, integrated assembly, which was looped around the pedicle base to ensure full circumferential contact to facilitate a precise and secure hot-snare resection ( Fig. 3b ). Subsequently, a third snare was applied to constrict the assembly, ensuring secure ligation ( Fig. 3c ). Hot snare resection was then performed ( Fig. 3d–e , Video 1 ). No intraprocedural or postprocedural adverse events (e.g., bleeding, perforation, or infection) occurred. Follow-up endoscopy at 3 months revealed complete mucosal healing with no evidence of the luminal stricture ( Fig. 3f ), and the patient remained asymptomatic. Given the large size of the resected lesion, en bloc transoral retrieval was not feasible. We therefore extracted the specimen in a piecemeal fashion and reconstructed ex vivo to verify its intact morphology ( Fig. 4 ). Histopathological analysis confirmed a well-differentiated liposarcoma (WDLPS).

**Video 1** Demonstration of the endoscopic resection of a 21-cm giant well-differentiated liposarcoma using a modified snare technique.


Esophageal WDLPSs are rare pedunculated lesions typically arising from the restricted postcricoid region, with therapeutic options ranging from surgical resection to various endoscopic interventions. 1​ 2​ 3​ ​4​ For such broad-based lesions, conventional ESD often carries a high risk of anatomical disorientation, while peroral protrusion remains unfeasible. Our modified snare technique offers a safe, cost-effective, and reproducible alternative that simplifies the management of broad-pedicled challenging giant lesions.

Endoscopy_UCTN_Code_TTT_1AO_2AG_3AB.
